# Exosome-Like Vesicles as New Mediators and Therapeutic Targets for Treating Insulin Resistance and *β*-Cell Mass Failure in Type 2 Diabetes Mellitus

**DOI:** 10.1155/2019/3256060

**Published:** 2019-03-12

**Authors:** Qian Ge, Xin Xin Xie, Xiaoqiu Xiao, Xi Li

**Affiliations:** ^1^Department of Endocrinology, The First Affiliated Hospital of Chongqing Medical University, 400016 Chongqing, China; ^2^The Biology Science Institutes, Chongqing Medical University, Chongqing 400016, China; ^3^The Chongqing Key Laboratory of Translational Medicine in Major Metabolic Diseases, The First Affiliated Hospital of Chongqing Medical University, 400016 Chongqing, China

## Abstract

Exosome-like vesicles (ELVs), the smallest class of extracellular vesicles released from cells, function in cellular crosstalk and therefore profoundly affect physiologic responses and pathologic progression. A growing body of evidence supports a novel role for ELVs as important mediators and therapeutic targets due to their effects on regulation of both insulin signaling and *β*-cell mass. Pathologic conditions associated with type 2 diabetes (such as high blood glucose, inflammation, hypoxia, and fatty acids) can alter the quantity and components of ELVs secreted from the pancreas or peripheral insulin-targeting tissues. These released ELVs can either enter the blood circulation or be taken up by neighboring cells or macrophages, which can lead to insulin resistance or *β*-cell apoptosis. This review focuses on the roles of ELVs in insulin resistance and *β*-cell failure and also highlights the potential use of ELVs and exosome-based delivery systems in therapeutic interventions aimed at treating type 2 diabetes mellitus as well as the challenges associated with exosome-targeting therapeutics.

## 1. Introduction

Diabetes mellitus is a major cause of morbidity and mortality and represents a serious threat to global human health and welfare. Type 2 diabetes mellitus (T2DM) accounts for 95% of diabetes cases and is characterized by peripheral insulin resistance (IR) and progressive pancreatic *β*-cell failure induced primarily by apoptosis of *β*-cells [1]. The mechanisms underlying the development of IR and *β*-cell apoptosis remain unclear, however. Most pharmacologic interventions used in the treatment of T2DM rely on modulating glycemia by promoting insulin secretion or supplementation.

Exosome-like vesicles (ELVs) are the smallest class of extracellular vesicles, and interest in their potential use in therapeutic applications is increasing. ELVs function in organ crosstalk and thus profoundly affect both normal physiologic processes and pathologic progression. Increasing evidence indicates that ELVs play functional roles in various cancers [2], neurologic disorders [3], metabolic diseases [4], autoimmune diseases [5], and cardiovascular diseases [6].

## 2. Biogenesis of ELVs

**Figure 1:**
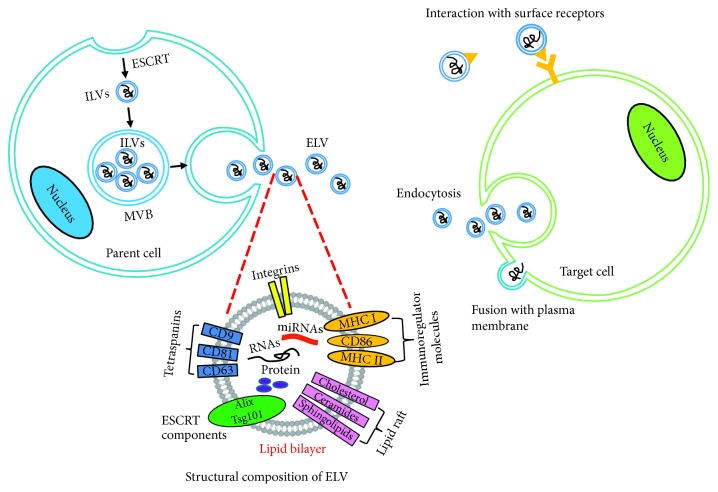
Schematic illustration of formation, structure, and uptake of exosome-like vesicles (ELVs). ILVs: intraluminal vesicles; MVB: multivesicular bodies; MHC: histocompatibility complex; ESCRT: endosomal sorting complexes required for transport, adapted from [9].

ELVs are released by virtually all cell types, and they range in diameter from 30 to 100 nm. ELVs are initially formed in intraluminal vesicles (ILVs) that group together to form larger membranous vesicles called multivesicular bodies (MVBs). MVBs are released into the extracellular space upon fusion with the plasma membrane. A variety of different pathways mediate MVB release. The most thoroughly characterized pathway involves the recruitment of endosomal sorting complexes required for transport (ESCRT) [7]. These complexes recruit proteins for internalization (e.g., ubiquitinated proteins and clathrin), initiating the budding process and driving membrane invagination and separation [8]. ELVs contain bioactive proteins, lipids, and a broad range of nucleic acids, such as genomic DNA, mRNA, and small noncoding RNAs. Although the mechanisms by which these molecules are incorporated into ELVs remain unclear, the composition of ELVs reflects the molecules present in their parent cells. The most abundant proteins present in ELVs include members of the tetraspanin family (CD9, CD63, and CD81) and ESCRT proteins (TSG101, ALIX) [9]. These proteins have been used as markers for identifying purified ELVs [10]. When ELVs fuse with recipient cells, they release their contents into the cytoplasmic space, which enables the horizontal transfer of their contents. Thus, ELVs can function as essential messengers. The uptake of ELVs by recipient cells is an energy-dependent active process involving membrane receptor recognition, adhesion molecules that induce fusion and endocytosis, and phagocytosis. Upon internalization, the recipient cell responds to the transferred exosome contents by modulating its basal function and gene expression [11] (Figure 1).

## 3. Effect of ELVs on Pancreatic *β*-Cell Mass

Both type 1 and type 2 diabetes are associated with a significant deficit in *β*-cell mass (65% in type 2 diabetes, 99% in type 1 diabetes) [1, 12], and this deficit plays a particularly pivotal role in the pathogenesis of type 2 diabetes. The major defect leading to a decrease in *β*-cell mass in type 2 diabetes is an increase in apoptosis [1]. As such, preserving residual *β*-cell mass is fundamental in the therapeutic management of type 2 diabetes.

Recent studies have identified a novel ELV-mediated signaling mechanism between pancreatic *β*-cells. Guay et al. found that ELVs isolated from the culture medium of MIN6B1 *β*-cells and islets of mice, rats, or humans contain microRNAs (miRNAs) known to be expressed at high levels in *β*-cells, such as miR-7, miR-29a, and miR-146a. Moreover, exposure of *β*-cells to inflammatory cytokines alters the composition of several miRNAs in ELVs. Incubation of untreated MIN6B1 or mouse islet *β*-cells in the presence of miRNA-containing ELVs isolated from the culture medium of cytokine-treated MIN6B1 cells triggers apoptosis of recipient *β*-cells [13]. miR-106b-5p and miR-222-3p contained in mouse serum ELVs contribute to bone marrow transplantation-induced *β*-cell regeneration by increasing the proliferation of residual *β*-cells. Systemic administration of miR-106b and miR-222 in mice significantly lowers fasting blood glucose and enhanced fasting insulin levels [14].

**Figure 2:**
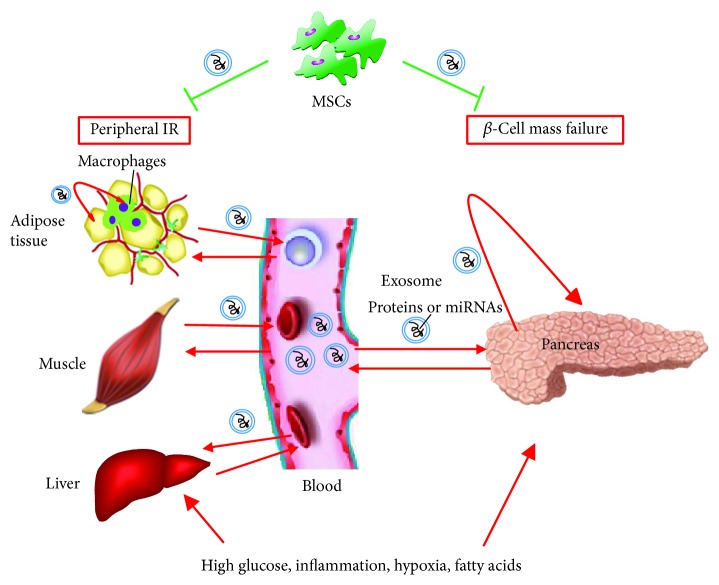
Schematic illustration of how exosome-like vesicles (ELVs) serve as mediators and therapeutic targets for treating insulin resistance (IR) and *β*-cell mass failure in type 2 diabetes. Type 2 diabetes-associated pathologic conditions (such as high glucose, inflammation, hypoxia, and high fatty acids) modify the quantity and components of ELVs secreted from the pancreas or peripheral insulin-targeting tissues. These released ELVs may either enter into the circulation or enter neighboring cells or macrophages, delivering IR or *β*-cell apoptosis signatures. ELVs released by mesenchymal stem cells (MSCs) can improve insulin sensitivity and enhance *β*-cell proliferation. Red arrows indicate stimulation or activation; green blunted arrows indicate inhibition.

High blood glucose levels can alter the components of ELVs secreted from rat pancreatic *β*-cells with increased exosomal miR-15a [15]. In addition to miRNAs, *β*-cells secrete various exosomal proteins. Cianciaruso et al. found that exposure of rat islets to certain cytokines (interleukin- (IL-) 1*β* and interferon-*γ*) upregulates the expression of immunomodulatory proteins involved in the endoplasmic reticulum (ER) stress response in rat islet ELVs. Moreover, rat and human islet cells release GAD65, IA-2 (an intracellular *β*-cell autoantigen in human type 1 diabetes), and proinsulin via ELVs, which are taken up by and activate antigen-presenting cells, autoreactive marginal zone-like B cells, and T lymphocytes, contributing to the immune response associated with diabetes [16]. Interestingly, ELVs have been shown to be effective vehicles for the delivery of target proteins into *β*-cells. Tang et al. packaged ceramidase into ELVs by overexpressing this protein in cultured INS-1 cells. The exosome-packaged ceramidase protected *β*-cells from palmitate-induced apoptosis [17] (Figure 2).

## 4. Role of ELVs in Insulin Resistance (IR)

IR, which is caused by deficits at several levels of the insulin signaling pathway, is the major feature of type 2 diabetes and also commonly associated with the development of hypertension and atherosclerosis. Hence, improving insulin sensitivity without increasing the risk of hypoglycemia and cardiovascular disease is the major aim of T2DM treatment, particularly in obese T2DM patients [18].

### 4.1. Role of Adipose Tissue-Derived ELVs in IR

Adipose tissue plays a key role in the development of IR through secretion of proinflammatory adipokines and miRNAs [19, 20]. Recent studies reported that adipose tissue is also a major source of circulating exosomal miRNAs [21], which may represent additional vehicles for communication between adipose tissue and other metabolic organs in the regulation of systemic IR. Adipocyte-derived ELVs also contain the adipose-specific protein markers fatty acid-binding protein (FABP-4) and adiponectin, as well as a number of proinflammatory adipokines, including tumor necrosis factor alpha (TNF-*α*), macrophage colony-stimulating factor (MCSF), interleukin-6 (IL-6), monocyte chemoattractant protein-1 (MCP-1), macrophage migration inhibitory factor (MIF), and retinol-binding protein 4 (RBP-4) [22, 23]. Kranendonk et al. showed that the number of omental adipose tissue-derived ELVs is positively correlated with homeostasis model assessment (HOMA) IR.

Obesity-induced low-grade inflammation is known to be an important mechanism linking obesity to IR. One study demonstrated that ELVs released by human adipocytes differentiated *in vitro* or from adipose explants cultured ex vivo altered the gene expression of recipient human blood monocytes toward to a phenotype of human adipose tissue macrophages, which serves as a major inducer of IR. Indeed, conditioned medium of macrophages treated with ELVs was shown to inhibit phosphorylation of human adipocyte Akt (a key kinase in insulin signaling) [22]. An *in vivo* experiment further confirmed that the intravenous injection of ELVs released in adipose tissues of obese mice results in IR in recipient lean mice via activation of macrophages with increased secretion of TNF-*α* and IL-6 [23]. Exosomal miR-155 released by adipose tissue macrophages of obese mice promotes IR in lean mice via a decrease in insulin-stimulated phosphorylation of Akt and expression of peroxisome proliferator-activated receptor *γ* (PPAR*γ*) in skeletal muscle, the liver, and adipose tissue [24]. A recent study reported that 3T3-L1 adipocyte-derived ELVs carrying sonic hedgehog protein induced M1 polarization of bone marrow-derived macrophages via the Ptch and PI3K signaling pathways and the adipocyte-derived ELVs downregulated the expression of insulin receptor substrate-1 (IRS-1) and hormone-sensitive lipase (HSL) in adipocytes [25].

Adipocyte hypoxia caused by excessive expansion of adipose tissue is another mechanism leading to IR. Mleczko et al. showed that ELVs released by hypoxic adipocytes impaired insulin-stimulated 2-deoxyglucose uptake and reduced insulin-mediated phosphorylation of Akt in recipient adipocytes, suggesting that ELVs mediate the transfer of hypoxia-induced IR signatures within adipose tissue [26].

Interactions between adipose ELVs and muscle and liver cells have also been reported. Adipocyte-derived exosomal miR-27a is taken up by C2C12 skeletal muscle cells and impairs local insulin signaling via repression of PPAR*γ* [27]. ELVs secreted by both human subcutaneous adipose tissue and visceral adipose tissue impair HepG2 hepatocyte insulin signaling by inhibiting insulin-stimulated Akt phosphorylation [28].

### 4.2. Role of Muscle-Derived ELVs in IR

In addition to adipose tissue, skeletal muscle is also an active endocrine organ, secreting a number of the so-called “myokines” [29]. ELVs released by C2C12 cells exposed to palmitate and from muscle tissue of mice with palm oil-induced IR increased the total content of Akt in recipient myotubes but had no effect on insulin-stimulated Akt phosphorylation [30]. This finding indicated that muscle cell-derived ELVs do not transfer lipid-induced IR between cells. However, ELVs do transfer lipid-induced IR between muscle cells and pancreatic *β*-cells. ELVs from muscle tissue of mice with lipid-induced IR were taken up by *β*-cells *in vivo*. *In vitro* experiments further demonstrated that muscle ELVs from IR mice modulate the expression of a significant number of genes in MIN6B1 cells, leading to proliferation of these cells [31]. This ELV-mediated crosstalk between muscle IR and *β*-cell proliferation could represent a novel mechanism that explains the compensatory enlargement in *β*-cell mass during IR. Elucidation of the mechanism underlying compensatory expansion of *β*-cells may shed new light on therapeutic strategies for preserving *β*-cell mass in T2DM (Figure 2).

### 4.3. Role of Circulating ELVs in IR

Evidence increasingly demonstrates that the cargo of circulating ELVs plays an important role in IR. An important recent finding showed that circulating exosomal miRNAs are essential for maintaining systemic glucose and insulin homeostasis. Fat-specific inhibition of miRNA production results in marked decreases in circulating exosomal miRNAs and development of glucose intolerance and IR, whereas restoration of circulating miRNAs by implanting adipose tissue into fat-specific miRNA knockout mice was shown to improve glucose tolerance and reduce IR [21]. Freeman et al. found that ELV secretion in human plasma increases during IR. Using an ELISA method, they identified 12 phosphorylated proteins (e.g., phospho-Akt, phospho-insulin receptor, and phospho-IRS1) involved in insulin signaling pathways in circulating human ELVs. Among these proteins, levels of the phosphorylated insulin receptor were decreased in ELVs of individuals with IR. Levels of phospho-S6RP, phospho-GSK3*β*, and phospho-Akt were inversely correlated with HOMA *β*-cell function, and levels of phospho-S6RP were inversely correlated with HOMA IR [32]. Another clinical study showed that changes in circulating exosomal miRNAs in obese subjects who underwent gastric bypass surgery are closely correlated with decreasing IR. This finding provided evidence that ELVs could serve as prognostic markers [33]. Other research demonstrated that circulating ELVs impair insulin signaling *in vitro*, as ELVs isolated from plasma of obese women induced a decrease in insulin-mediated 2-deoxyglucose uptake in cultured 3T3-L1 adipocytes [26] (Figure 2).

## 5. Role of Mesenchymal Stem Cell- (MSC-) Derived ELVs in Diabetes

MSCs have emerged as cell factories for ELV production in the laboratory. Similar to MSCs, ELVs released by MSCs have a strong capacity for enhancing cell proliferation, reducing apoptosis, and suppressing inflammatory and/or immune responses. MSC-released ELVs represent a safe and effective cell-free therapy for treating myocardial infarction, acute kidney injury, and liver injury. The potential use of MSC-derived ELVs in treatment of diabetes has also been investigated. Zhao et al. showed that ELVs obtained from adipose-derived stem cells (ADSCs) induce M2 polarization in macrophages *in vitro*, with increases in mRNA levels of M2-related arginase-1 (Arg-1) and interleukin-10 (IL-10). This M2 polarization was also observed in adipose tissue macrophages *in vivo* by intraperitoneal administration of ADSC-derived ELVs in diet-induced obese mice, which resulted in attenuated adipose tissue inflammation. The administration of ADSC-derived ELVs was also shown to improve systemic glucose tolerance and insulin sensitivity and attenuate adipocyte hypertrophy [34]. In another study, Sun et al. examined ELVs isolated from human umbilical cord (huc) MSCs. They found that intravenous injection of hucMSC-derived ELVs into fat-fed/streptozotocin- (STZ-) induced rats with T2DM attenuated both hyperglycemia and IR. This insulin-sensitive effect of hucMSC-derived ELVs was associated with their ability to increase the insulin-stimulated phosphorylation of insulin receptor substrate-1 (IRS-1) and Akt in cell models of IR. Furthermore, intravenously injected hucMSC-derived ELVs promoted insulin secretion and islet regeneration by inhibiting STZ-induced *β*-cell apoptosis [35] (Figure 2).

## 6. Diagnostic and Therapeutic Potential of ELVs

The observations that ELVs are largely dysregulated in diabetes and that exosomal cargo components regulate *β*-cell function and insulin signaling in recipient cells make them attractive therapeutic targets in treating T2DM.

### 6.1. ELVs as Diagnostic Tools

Circulating ELVs have been proposed as novel biomarkers of various diseases for a number of reasons. First, in contrast to signaling molecules in body fluids, the intravesicle cargo is remarkably stable, as it is protected from degradation by the ELV lipid bilayer. Second, ELVs are enriched in certain proteins, lipids, and RNAs and lacking in others, which can reflect parent cell characteristics under various pathologic conditions. Third, ELVs are very abundant in the circulation and can be therefore analyzed using small volumes of frozen serum/plasma samples.

Multiple reports have noted the potential clinical use of blood ELVs for the diagnosis of cancer. Shao et al. established a method using a microfluidic platform. Their method, known as immunomagnetic exosomal RNA analysis, enables the enrichment of cancer-specific ELVs from blood and rapid, on-chip analysis of exosomal mRNA content. Using this technique, the authors identified mRNAs of two key enzymes within circulating ELVs. As the exosomal mRNA levels of these two enzymes correlated well with the levels in parental cells and the levels changed markedly during treatment, these exosomal mRNAs could be used as markers to predict drug response in glioblastoma patients [36]. This application can also be extended to other metabolic disorders. For example, the capture of *β*-cell-specific ELVs and subsequent analysis of their intravesicular cargo could provide insights into the function of *β*-cells in diabetes patients and aid in predicting the efficacy of antidiabetes drugs in order to achieve better clinical outcomes.

### 6.2. ELVs as Therapeutic Tools

The finding that ELVs can be taken up by cells with low possibility of immune rejection has spurred research into the use of ELVs as natural drug delivery tools. For therapeutic purposes, ELVs can be naturally generated by MSCs, constructed by modifying genes of donor cells, decorated by directly loading exogenous molecules, or artificially synthesized. As reviewed above, MSC-derived ELVs can be injected directly into the blood and have exhibited protective effects in diabetes treatment. Given the possibility that not all components of ELVs are required for their function, exosomal components could be specifically tailored in response to genetic modifications of cells. Therapeutic nucleic acids or proteins or their inhibitors could be loaded into ELVs by overexpressing these molecules in the donor cells. Research indicates that after uptake by recipient cells, these exogenous molecules are functionally active and exert the expected effects. Exogenous nucleic acids or proteins could also be directly loaded into purified ELVs by electroporation, lipofection, sonication, or calcium chloride treatment. ELVs loaded with siRNA by electroporation can reportedly knockdown specific genes in recipient cells without inducing an obvious immune response [37]. Artificially synthesized exosome-mimics that contain only crucial components of natural ELVs have also been developed as more promising delivery systems. These exosome-mimics include liposomes [38] and exosome-mimetic nanovesicles [39]. The latter can be produced by extruding living cells through microsized filters [40, 41].

### 6.3. Potential Challenges in Exosome-Based Therapy

Although significant advances have been made in exosome-based therapy, various challenges remain to be overcome before routine clinical use. First, the cargos of ELVs are complex and thus difficult to thoroughly characterize. As such, it is possible that uncharacterized factors could induce undesired effects when used in therapeutic interventions. Second, biological fluids contain a mixture of ELV populations from various cell types. The identification of a single population of cell-specific vesicles (e.g., *β*-cell-derived ELVs) remains difficult. A third critical challenge involves the site-specific delivery of ELVs *in vivo*. Systemic intravenous delivery of ELVs has been achieved in treatments for cancer and liver diseases; however, efforts to target other specific tissues have been less successful due to the absence of cell-specific uptake. The decoration of ELVs by conjugating targeting moieties to therapeutic cargo to improve cell-specific delivery may be a more rational approach for managing local abnormalities with greater efficiency and precision and with fewer systemic side effects.

## 7. Conclusions

Current data highlight the importance of ELVs as mediators and therapeutic targets in the treatment of T2DM. The pathology associated with T2DM leads to modifications of exosomal components. The modified cargos of ELVs can be delivered to neighboring and/or distant cells, resulting in impaired *β*-cell mass and function and insulin signaling. MSC-released ELVs, by contrast, may exert protective effects in this regard.

The discovery that ELVs are remarkably stable and closely correlated with crucial metabolic parameters suggests that plasma ELVs could be useful diagnostic tools. The establishment of an exosome-based delivery system would facilitate their application in the management of T2DM. Despite considerable challenges confronting efforts to develop exosome-targeting therapeutics, especially regarding the development of methods for cell-specific delivery of targeting ELVs *in vivo*, the development of exosome-based therapeutics for diabetes treatment is warranted because of the serious public health threat posed by diabetes and related complications.
